# Dynamic associations of cholinesterase inhibitors and memantine with cognitive trajectories in individuals with Alzheimer’s or mixed dementia: a real-world analysis using the quality registry SveDem

**DOI:** 10.1186/s13195-025-01918-0

**Published:** 2025-11-28

**Authors:** Cen Chen, Minjia Mo, Madeleine Åkerman, Sara Garcia-Ptacek, Hong Xu, Maria Eriksdotter

**Affiliations:** 1 Department of Neurobiology, Care Sciences and Society, Division of Clinical Geriatrics, Karolinska Institutet, NEO, Blickagången 16, Huddinge, Stockholm, 141 52 Sweden; 2 Theme Inflammation and Aging, Karolinska University Hospital, Hälsovägen 13, Blickagången 20, Huddinge, Stockholm, 141 57 Sweden

**Keywords:** Cholinesterase inhibitors, Memantine, Time-varying medications, Alzheimer's disease and mixed dementia, MMSE

## Abstract

**Background:**

Alzheimer’s disease (AD) and mixed dementia (MxD) represent major public health concerns, yet there is limited real-world evidence on the long-term associations of commonly prescribed pharmacological treatments, particularly cholinesterase inhibitors (ChEIs) and their combination with memantine. This study aims to evaluate the long-term, time-varying associations of ChEIs and memantine on cognitive decline in a large, nationwide cohort of individuals diagnosed with AD or MxD.

**Methods:**

This observational study utilized data from the Swedish registry for cognitive/dementia disorders (SveDem), analyzing 32,282 individuals diagnosed with AD or MxD between 2007 and 2022. Patients were followed for up to 11 years to track treatment patterns and cognitive trajectories. Initiation of ChEIs (donepezil, galantamine, or rivastigmine) or memantine within six months after an AD or MxD diagnosis and then the time-varying medications (ChEIs alone, memantine alone or memantine added on to ChEIs) within six months after each follow-up were analyzed. Linear mixed-effects model was used to assess cognitive decline measured by Mini-Mental State Examination (MMSE) score trajectories.

**Results:**

Of the 32,282 participants (mean age 78.4 years; 61% women), 78.4% initiated treatment with ChEIs alone, and 21.6% with memantine alone. Over time, prescription patterns shifted from monotherapy to combination therapy, with donepezil and galantamine more likely to achieve 1 Defined Daily Dosages (DDD) than rivastigmine users. Memantine alone users experienced a yearly cognitive decline of 1.79 MMSE points (95% CI: −1.85, −1.73). Compared with memantine users, patients on ChEIs alone declined 0.65 points less per year (95% CI: −0.59, −0.72), while those on combination therapy declined 0.19 points less per year (95% CI: −0.10, −0.28). Among ChEIs, donepezil users experienced an annual decline of 1.02 points (95% CI: −1.06, −0.98). In comparison, galantamine users declined an additional 0.05 points per year (95% CI: −0.11, 0.01), and rivastigmine users declined an additional 0.17 points per year (95% CI: −0.24, −0.11), relative to donepezil.

**Conclusions:**

In this large cohort of patients with AD or MxD, ChEIs users alone, particularly donepezil and galantamine, showed slower cognitive decline compared to users of memantine or combination therapy users. While differences were modest, the results contribute to a better understanding of treatment trajectories in routine clinical practice.

**Supplementary Information:**

The online version contains supplementary material available at 10.1186/s13195-025-01918-0.

## Background

Alzheimer’s disease (AD) is a progressive neurodegenerative disorder characterized by cognitive decline, memory loss, and functional impairment. As the leading cause of dementia worldwide, AD represents a major public health challenge [1]. More than 50% of AD patients exhibit co-morbid vascular contributions to cognitive impairment and dementia, a condition referred to as mixed dementia (MxD) [2–4]. 

First-line pharmacological treatments for AD include the cholinesterase inhibitors (ChEIs), i.e. donepezil, galantamine, or rivastigmine, and the N-methyl-d-aspartate (NMDA) receptor antagonist memantine. These treatments have shown efficacy in improving cognition, psychotic symptoms, and daily functioning in randomized double-blind controlled trials (RCTs) involving individuals with AD[5–8], as well as in patients diagnosed with vascular dementia (VD) [9, 10]. However, while these clinical trials have demonstrated efficacy, they are often conducted under highly controlled conditions, with strict inclusion criteria and relatively short follow-up periods (from 3 to 6 months and a few up to 24 months), which may not fully reflect the long-term real-world outcomes in broader, among more heterogeneous patient populations.

Observational studies provide the advantage of evaluating the long-term effectiveness and safety of drugs in more diverse, non-selected patient populations. Despite this, only a few observational studies have explored the impact of ChEIs on cognitive decline in AD or other forms of dementia over extended periods, with the longest follow-up lasting up to 10 years[11–16]. These studies mainly focused on fixed exposures with less focus on the dynamic nature of real-world clinical practice where patients often change medications over time for various reasons.

There are numerous clinical short-term trials on the effectiveness of ChEIs and memantine, although few provide head-to-head comparisons of the different medications[5, 17]. Comparisons between the different ChEIs—donepezil, galantamine, and rivastigmine—are of particular interest, as the efficacy of these drugs may vary, and understanding which treatment provides the most sustained cognitive benefits could inform personalized treatment strategies. Furthermore, in clinical practice, patients often modify their treatment regimens over time, either switching between different ChEIs or combining ChEIs with memantine. The combination of ChEIs and memantine has shown promise as a synergistic therapeutic strategy, potentially offering more comprehensive management of cognitive symptoms by targeting both the cholinergic and glutamatergic systems [18]. However, the long-term outcomes of such combined treatments remain understudied, especially in clinical routine patient populations with AD or MxD.

Given the complexity and progressive nature of AD, as well as the evolving patterns of pharmacological treatment in real-world settings and the recent new disease-modifying dementia drugs[19, 20], evaluating the long-term, time-varying trajectories of currently approved treatments (ChEIs and memantine) is essential. Such analyses will provide a more accurate understanding of how these medications influence disease progression over time, accounting for treatment adjustments and patient variability. Moreover, ChEIs have also shown efficacy in Lewy body dementia[16], underscoring their broader therapeutic relevance across multiple dementia subtypes.

The objective of the present study was to assess the long-term, dynamic trajectories of ChEIs and memantine in a large cohort of patients with AD or MxD, with long-term follow-up data. In addition, to provide new insights into the real-world trajectories of these treatments, with the aim to contribute to the optimization of therapeutic strategies for managing dementia.

## Methods

### Data source

We analyzed data from the Swedish registry for cognitive/dementia disorders (SveDem; www.svedem.se*)*, a nationwide web-based quality register established in May 2007 to enroll and follow patients with dementia in Sweden. Data entry is performed online by clinical personnel directly involved in patient care, primarily physicians and dementia nurses at memory clinics, primary care centers, and hospitals, at the time of diagnosis and at follow-up. SveDem collects data on patient demographics, Mini-Mental State Examination (MMSE) scores, dementia subtype, and treatment information at baseline and at subsequent follow-up visits, which are scheduled annually but may vary in practice. For this study, SveDem was linked to several nationwide health registers: the National Patient Registry, which provides information on comorbidities diagnosed in specialist and hospital care; the Prescribed Drug Registry, which records all dispensed prescribed medications since 2006; and the Cause of Death Registry.

### Study population

We aimed to compare cognitive trajectories in patients with clinically diagnosed AD or MxD in clinical routine settings in Sweden who initiated anti-dementia treatment within six months of diagnosis and during subsequent follow-ups. Biomarker confirmation was not available for the majority of the patients and was not a requirement. The treatments considered were ChEIs alone or memantine alone within six months after the AD or MxD diagnosis, and the time varying medications (ChEIs or memantine or memantine added on to ChEIs) during follow-up. Patient selection flow chart was shown in supplementary Fig. 1. We included individuals diagnosed with AD or MxD (*N* = 53 185) registered between May 1 st, 2007 and May 7th, 2022 in SveDem, and up to four follow-ups for each participant. Individuals who died (*N* = 1 394) or ended the follow-up (*N* = 1 462) within the first six months after the dementia diagnosis were excluded, this criterion ensured that medication use could be ascertained, as pharmacological treatment was defined within 6 months of diagnosis, and that patients had at least the potential to contribute longitudinal MMSE data for trajectory analyses. Additional 1 989 individuals were excluded because of 0 or missing MMSE scores at the time of diagnosis. Individuals who were already on either ChEIs or memantine before the time of their dementia diagnosis (prevalent users, *N* = 6 271), as well as those who started concomitantly with both therapies (combination therapy users, *N* = 2 790) and those who did not use ChEIs nor memantine (no treatment, *N* = 6 829) within six months after the AD or MxD diagnosis were also excluded. Individuals who used memantine within six months after the AD or MxD diagnosis and added on ChEIs within six months after follow-ups (*N* = 168) were excluded because adding ChEIs after starting memantine might not align with clinical protocols. After applying our inclusion and exclusion criteria, the final sample consisted of 32 282 incident AD or MxD individuals, among whom 25 318 (78%) were ChEIs users and 22% memantine users within six months after the AD or MxD diagnosis.

**Fig. 1:**
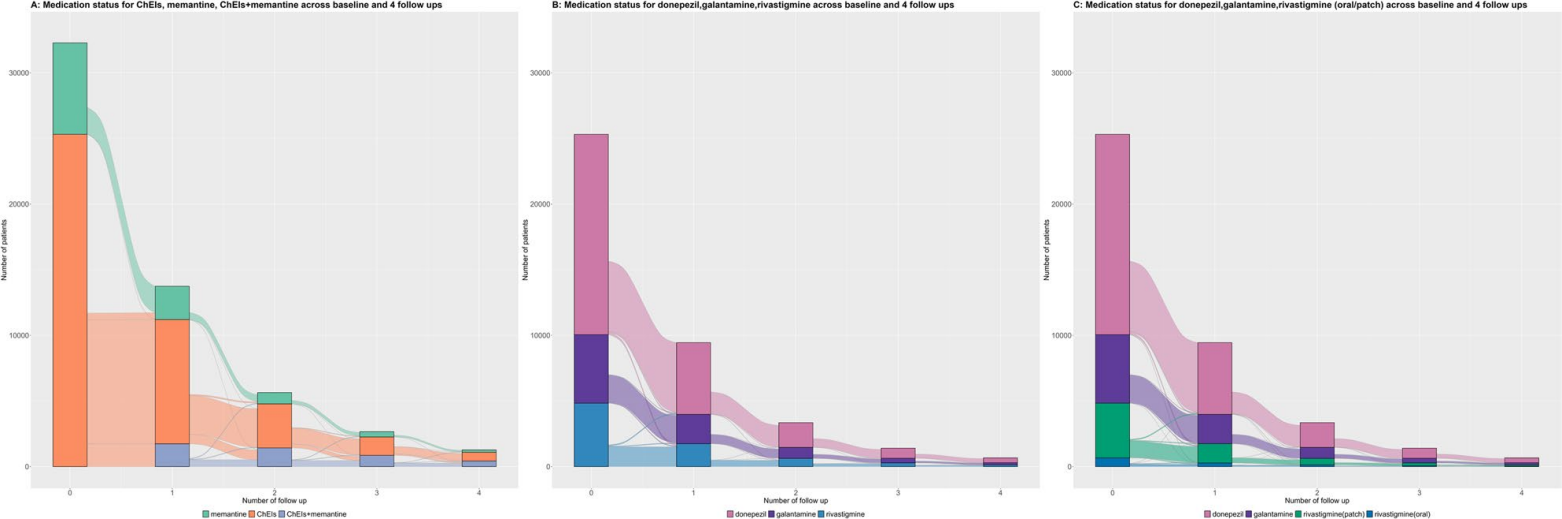
Sankey plot by treatment status: (**A**) Memantine, ChEIs, and ChEIs add on memantine, (**B**) Donepezil, galantamine, rivastigmine, (**C**) Donepezil, galantamine, rivastigmine (oral) and rivastigmine (patch)

### Exposure

Study exposure was the initiation of ChEIs (donepezil, rivastigmine, or galantamine) therapy alone or memantine alone within six months after the dementia diagnosis and the time varying medications (ChEIs or memantine or memantine added on to ChEIs) within six months after each follow-up. All patients were included regardless of treatment discontinuation, switching, or add-on therapy, with time-varying medication use and MMSE data incorporated to model real-world cognitive trajectories.

### Covariates

Covariates were defined at the AD or MxD diagnosis date including sex, age at diagnoses, calendar year of diagnosis, MMSE score at diagnosis, Charlson Comorbidity Index (CCI including possible presence of myocardial infarction, congestive heart failure, peripheral vascular disease, cerebrovascular disease, chronic obstructive pulmonary disease, chronic other pulmonary disease, rheumatic disease, hemiplegia, diabetes without chronic complication, diabetes with chronic complication, renal disease, mild liver disease, severe liver disease, peptic ulcer disease, malignancy, metastatic solid tumors, AIDS), hypertension, atrial fibrillation, presence of ongoing medications (defined by medication use within six months before diagnoses date, including anxiolytics, stains, antipsychotics, antidepressants, angiotensin-converting enzyme inhibitors/angiotensin receptor blockers (ACEI/ARBs), β-blockers, calcium channel blocker) [21]. The definitions of comorbidities and medications are summarized in Supplementary Table 1.

### Outcomes

The outcome was the trajectories of MMSE scores which were obtained from the SveDem database. Participants were followed from their dementia diagnosis date until the occurrence of death, or the end of follow-up (May 7, 2022), whichever happened first.

### Statistical analyses

First, to investigate the time-varying patterns of ChEIs and memantine usage in our cohort, we utilized a Sankey diagram to visualize the flow of medication use status from the diagnosis date through subsequent follow-ups. For each ChEIs, prescription trends were further analyzed by calculating the proportion of patients who achieved the Defined Daily Dosages (DDD) within six months after the diagnosis date and each follow ups. The DDDs represent the assumed average maintenance dose per day for a drug used for its main indications in adults. The value of DDD is established by the World Health Organization International Working Group for Drug Statistics Methodology and is a widely accepted method for measuring drug utilization [22]. 1 DDD is equal to donepezil 7.5 mg, oral rivastigmine 9 mg, rivastigmine patch 9.5 mg or galantamine 16 mg.

Second, for MMSE score trajectories, we applied linear mixed-effects models with random intercept to incorporate individuals with at most 4 visits throughout 11 years follow up due to very few observations after 4 follow-ups. The mixed effects model included treatments, follow-up year, and the interaction between treatment and follow-up year to estimate the differences in the annual MMSE change attributed to different treatments, with all the covariates adjusted. Furthermore, we considered the potential impact of attrition due to dropout or the presence of a competing risk (e.g., death) during the follow-up. To address this concern, we applied inverse probability of censoring weighting in conjunction with the mixed-effect model. This approach allowed us to appropriately handle potential biases arising from attrition in our analysis.

Third, we also evaluated the consistency of associations across ChEIs types (donepezil, galantamine, or rivastigmine) with MMSE score trajectories. Additionally, since rivastigmine was administered both orally or via transdermal patch, we further examined MMSE score trajectories separately for patients receiving oral rivastigmine and those using the rivastigmine patch.

### Sensitivity analysis and subgroup analysis

We repeated the analysis incorporating up to two or seven follow-up visits per participant, to assess the impact of potential attrition on the consistency of associations when observations are lost at later follow-up intervals. We selected two and seven follow-up visits, as our data includes a maximum of seven follow-ups over a 13-year follow-up period. This approach enabled us to assess the robustness of the results across scenarios representing both minimal and maximal participant retention.

In addition, subgroup analyses were performed to test for potential effects modified by age (using median split; ≤78 vs. >78 years), sex (male vs. female), dementia type (AD vs. MxD) and baseline severity (very mild: baseline MMSE ≥ 25; mild 20–24; moderate 10–19; severe ≤ 9) on the association between treatment and MMSE trajectories.

Statistical analyses were conducted with R 4.2.1, with statistical tests using a 2-tailed *P* < 0.05 as the level of statistical significance.

## Results

### Patient characteristics

We included a total of 32 282 individuals (61.4% women) with incident AD or MxD (35.1%) between May 1, 2007, and May 7, 2022 who started on treatment with ChEI or memantine within 6 months after the dementia diagnosis date. The mean age at diagnoses was 78.4 ± 7.6 years and the mean MMSE score was 21.4 ± 4.7 points. Among this cohort, 25 318 individuals started on ChEIs alone, and 6 964 individuals started on memantine alone. Users of ChEIs were younger, had higher MMSE scores, and lower CCI compared to memantine users. Among the ChEIs users, those on donepezil were more numerous, older, and had lower MMSE scores than those on galantamine or rivastigmine. The characteristics of the study participants are detailed in Table 1 and Table 2.

**Table 1:** Baseline characteristics stratified by treatment status (memantine, ChEIs) within 6 months after incident diagnosis of AD or MxD

characteristics		overall	memantine	ChEIs	*P*
*n*		32,282	6964	25,318	
Sex (%)	Male	12,472 (38.6)	2967 (42.6)	9505 (37.5)	< 0.001
	Female	19,810 (61.4)	3997 (57.4)	15,813 (62.5)	
Age at diagnoses (mean (SD))		78.4 (7.6)	80.8 (6.6)	77.8 (7.8)	< 0.001
Age group at diagnoses (%)	39–64	1591 (4.9)	91 (1.3)	1500 (5.9)	< 0.001
	65–74	7254 (22.5)	1092 (15.7)	6162 (24.3)	
	75–84	16,296 (50.5)	3651 (52.4)	12,645 (49.9)	
	85–94	7044 (21.8)	2092 (30.0)	4952 (19.6)	
	95–105	97 (0.3)	38 (0.5)	59 (0.2)	
Dementia type (%)	AD	20,958 (64.9)	3402 (48.9)	17,556 (69.3)	< 0.001
	MxD	11,324 (35.1)	3562 (51.1)	7762 (30.7)	
Baseline MMSE (mean (SD))		21.4 (4.7)	19.7 (5.2)	21.9 (4.4)	< 0.001
Baseline MMSE stages (%)	1–9	593 (1.8)	276 (4.0)	317 (1.3)	< 0.001
	10–19	9190 (28.5)	2857 (41.0)	6333 (25.0)	
	20–24	13,425 (41.6)	2524 (36.2)	10,901 (43.1)	
	25+	9074 (28.1)	1307 (18.8)	7767 (30.7)	
CCI (mean (SD))		0.9 (1.5)	1.2 (1.7)	0.8 (1.5)	< 0.001
Comorbidities					
Myocardial infarction (%)		2374 (7.4)	758 (10.9)	1616 (6.4)	< 0.001
Congestive heart failure (%)		2216 (6.9)	814 (11.7)	1402 (5.5)	< 0.001
Peripheral vascular disease (%)		1164 (3.6)	354 (5.1)	810 (3.2)	< 0.001
Cerebrovascular disease (%)		3637 (11.3)	1056 (15.2)	2581 (10.2)	< 0.001
Chronic obstructive pulmonary disease (%)		1302 (4.0)	434 (6.2)	868 (3.4)	< 0.001
Renal disease (%)		787 (2.4)	244 (3.5)	543 (2.1)	< 0.001
Malignancy (%)		4011 (12.4)	996 (14.3)	3015 (11.9)	< 0.001
Diabetes (%)		6053 (18.8)	1460 (21.0)	4593 (18.1)	< 0.001
Hypertension (%)		10,456 (32.4)	2869 (41.2)	7587 (30.0)	< 0.001
Atrial fibrillation (%)		3904 (12.1)	1322 (19.0)	2582 (10.2)	< 0.001
Medications					
Anxiolytics (%)		3572 (11.1)	928 (13.3)	2644 (10.4)	< 0.001
Statins (%)		10,610 (32.9)	2467 (35.4)	8143 (32.2)	< 0.001
Antipsychotics (%)		1206 (3.7)	407 (5.8)	799 (3.2)	< 0.001
Antidepressants (%)		8892 (27.5)	2019 (29.0)	6873 (27.1)	0.002
ACEI/ARBs (%)		12,001 (37.2)	2912 (41.8)	9089 (35.9)	< 0.001
Beta blockers (%)		10,654 (33.0)	2882 (41.4)	7772 (30.7)	< 0.001
Calcium channel blockers (%)		7105 (22.0)	1645 (23.6)	5460 (21.6)	< 0.001

As expected with a large cohort, nearly all group comparisons reached statistical significance (*p* < 0.001). These values largely reflect sample size rather than clinically meaningful differences, and the descriptive percentages and absolute differences provide the most relevant information for interpretation

*Abbreviations*:* ACEI*, angiotensin-converting enzyme inhibitors, *ARB *angiotensin receptor blockers, *CCI *Charlson Comorbidity Index

**Table 2:** Baseline characteristics stratified by treatment status (donepezil, galantamine, rivastigmine) within 6 months after incident diagnosis of AD or MxD

characteristics		overall	donepezil	galantamine	rivastigmine	rivastigmine (patch)	rivastigmine (oral)
*n*		25,318	15,266	5217	4835	4169	666
Sex (%)	Male	9505 (37.5)	5634 (36.9)	1957 (37.5)	1914 (39.6)	1632 (39.1)	282 (42.3)
	Female	15,813 (62.5)	9632 (63.1)	3260 (62.5)	2921 (60.4)	2537 (60.9)	384 (57.7)
Age at diagnoses (mean (SD))		77.8 (7.8)	78.8 (7.4)	76.2 (8.1)	76.3 (8.0)	76.1 (8.1)	77.5 (7.3)
Age group at diagnoses (%)	39–64	1500 (5.9)	608 (4.0)	490 (9.4)	402 (8.3)	367 (8.8)	35 (5.3)
	65–74	6162 (24.3)	3294 (21.6)	1489 (28.5)	1379 (28.5)	1213 (29.1)	166 (24.9)
	75–84	12,645 (49.9)	7857 (51.5)	2446 (46.9)	2342 (48.4)	1980 (47.5)	362 (54.4)
	85–94	4952 (19.6)	3462 (22.7)	785 (15.0)	705 (14.6)	603 (14.5)	102 (15.3)
	95–105	59 (0.2)	45 (0.3)	7 (0.1)	7 (0.1)	6 (0.1)	1 (0.2)
Dementia type (%)	AD	17,556 (69.3)	10,721 (70.2)	3539 (67.8)	3296 (68.2)	2831 (67.9)	465 (69.8)
	MxD	7762 (30.7)	4545 (29.8)	1678 (32.2)	1539 (31.8)	1338 (32.1)	201 (30.2)
Baseline MMSE (mean (SD))		21.9 (4.4)	21.6 (4.4)	22.3 (4.5)	22.4 (4.5)	22.3 (4.6)	22.5 (4.4)
Baseline MMSE stages (%)	1–9	317 (1.3)	188 (1.2)	67 (1.3)	62 (1.3)	56 (1.3)	6 (0.9)
	10–19	6333 (25.0)	4128 (27.0)	1133 (21.7)	1072 (22.2)	927 (22.2)	145 (21.8)
	20–24	10,901 (43.1)	6860 (44.9)	2106 (40.4)	1935 (40.0)	1677 (40.2)	258 (38.7)
	25+	7767 (30.7)	4090 (26.8)	1911 (36.6)	1766 (36.5)	1509 (36.2)	257 (38.6)
CCI (mean (SD))		0.8 (1.5)	0.9 (1.5)	0.6 (1.3)	0.9 (1.5)	0.9 (1.5)	0.9 (1.5)
Comorbidities							
Myocardial infarction (%)		1616 (6.4)	1054 (6.9)	249 (4.8)	313 (6.5)	260 (6.2)	53 (8.0)
Congestive heart failure (%)		1402 (5.5)	911 (6.0)	221 (4.2)	270 (5.6)	231 (5.5)	39 (5.9)
Peripheral vascular disease (%)		810 (3.2)	521 (3.4)	111 (2.1)	178 (3.7)	156 (3.7)	22 (3.3)
Cerebrovascular disease (%)		2581 (10.2)	1626 (10.7)	416 (8.0)	539 (11.1)	459 (11.0)	80 (12.0)
Chronic obstructive pulmonary disease (%)		868 (3.4)	581 (3.8)	132 (2.5)	155 (3.2)	124 (3.0)	31 (4.7)
Renal disease (%)		543 (2.1)	357 (2.3)	70 (1.3)	116 (2.4)	107 (2.6)	9 (1.4)
Malignancy (%)		3015 (11.9)	1935 (12.7)	473 (9.1)	607 (12.6)	536 (12.9)	71 (10.7)
Diabetes (%)		4593 (18.1)	2895 (19.0)	829 (15.9)	869 (18.0)	738 (17.7)	131 (19.7)
Hypertension (%)		7587 (30.0)	4792 (31.4)	1369 (26.2)	1426 (29.5)	1230 (29.5)	196 (29.4)
Atrial fibrillation (%)		2582 (10.2)	1681 (11.0)	404 (7.7)	497 (10.3)	424 (10.2)	73 (11.0)
Medications							
Anxiolytics (%)		2644 (10.4)	1598 (10.5)	533 (10.2)	513 (10.6)	444 (10.7)	69 (10.4)
Statins (%)		8143 (32.2)	5000 (32.8)	1552 (29.7)	1591 (32.9)	1350 (32.4)	241 (36.2)
Antipsychotics (%)		799 (3.2)	429 (2.8)	186 (3.6)	184 (3.8)	167 (4.0)	17 (2.6)
Antidepressants (%)		6873 (27.1)	3985 (26.1)	1475 (28.3)	1413 (29.2)	1226 (29.4)	187 (28.1)
ACEI/ARBs (%)		9089 (35.9)	5713 (37.4)	1717 (32.9)	1659 (34.3)	1425 (34.2)	234 (35.1)
Beta blockers (%)		7772 (30.7)	4802 (31.5)	1445 (27.7)	1525 (31.5)	1308 (31.4)	217 (32.6)
Calcium channel blockers (%)		5460 (21.6)	3468 (22.7)	1031 (19.8)	961 (19.9)	836 (20.1)	125 (18.8)

*Abbreviations*: *ACEI *angiotensin-converting enzyme inhibitors, *ARB *angiotensin receptor blockers,* CCI* weighted Charlson Comorbidity Index

### Time-varying patterns of cholinesterase inhibitors and memantine

The trajectories of medication status were visualized in the Sankey plots (Fig. 1). Among all the 32 282 individuals, 78.4% started with ChEIs and 21.6% started with memantine from the diagnosis date (Supplementary Table 2). Of all the 25 318 ChEIs users, 60.3% of them started with donepezil, 20.6% with galantamine and 19.1% with rivastigmine (16.5% with the rivastigmine patch, 2.6% with oral rivastigmine) (Supplementary Table 3).

During the first follow-up period (follow-up time 1 ± 0.6 years), the percentages of individuals using ChEIs or memantine alone were slightly decreased, while those with ChEIs who started to add on memantine increased. During the following follow-up periods, the proportion of individuals using ChEIs (78 to 52%) or memantine alone (22 − 14%) continued to decrease, and those with ChEIs with added on memantine treatment increased (0–34%) (Fig. 1A and Supplementary Table 2).

During the follow-ups, the proportion of use for each ChEIs is decreasing due to an increase in those on ChEIs where memantine is added, loss of follow up or death (Supplementary Table 3). The percentage of galantamine users increased over time, donepezil slightly decreased, rivastigmine slightly increased (Fig. 1B and Supplementary Table 3). Among the rivastigmine users, there was a slight decrease in the use of the patch formulation, accompanied by a modest increase in the use of the oral formulation, albeit at overall low numbers (Fig. 1C and Supplementary Table 3).

Specific transitions between baseline and follow-ups 1 and 2, 2 and 3, and 3 and 4 are shown in Supplementary Fig. 2.

### Prescription patterns of optimal doses of cholinesterase inhibitors

The proportion of patients who achieved 1 DDD of donepezil, galantamine, rivastigmine (oral), or rivastigmine (patch) within six months of the diagnosis date and each follow-up interval is shown in Table 3. Within 6 months after diagnosis, 4.5% of donepezil users had achieved 1 DDD, with this proportion increasing to 81.2% during follow-up. For galantamine users, 7.9% had reached 1 DDD within 6 months after diagnosis, rising to 91.7% over the follow-up period. None of the patients using oral rivastigmine reached 1 DDD neither within 6 months after diagnosis nor at follow-up. Among rivastigmine patch users, 4.2% reached 1 DDD within 6 months after diagnosis, with the proportion increasing to 84.7% during follow-up. Patients who initiated treatment later within the six-month period may have required additional time to achieve the optimal dose.

**Table 3:** The proportion of patients who achieved the recommended daily dose of donepezil, galantamine, Rivastigmine (oral), or Rivastigmine (patch) within six months after diagnosis date and at each follow-up interval

Treatment status	formulation	Medication	Number of patients below 1 DDD	Number of patients reach 1 DDD	Total patients	Proportion of patients who reached 1 DDD (%)
Within six months after diagnosis	oral	donepezil	14,577	689	15,266	4.5
Follow up 1	oral	donepezil	1605	3861	5466	70.6
Follow up 2	oral	donepezil	423	1461	1884	77.5
Follow up 3	oral	donepezil	138	623	761	81.9
Follow up 4	oral	donepezil	68	294	362	81.2
Within six months after diagnosis	oral	galantamine	4806	411	5217	7.9
Follow up 1	oral	galantamine	314	1922	2236	86.0
Follow up 2	oral	galantamine	93	747	840	89.0
Follow up 3	oral	galantamine	30	327	357	91.6
Follow up 4	oral	galantamine	14	154	168	91.7
Within six months after diagnosis	oral	rivastigmine	666	0	666	0
Within six months after diagnosis	patch	rivastigmine	3993	176	4169	4.2
Follow up 1	oral	rivastigmine	277	0	277	0
Follow up 1	patch	rivastigmine	435	1033	1468	70.4
Follow up 2	oral	rivastigmine	133	0	133	0
Follow up 2	patch	rivastigmine	93	400	493	81.1
Follow up 3	oral	rivastigmine	69	0	69	0
Follow up 3	patch	rivastigmine	31	183	214	85.5
Follow up 4	oral	rivastigmine	36	0	36	0
Follow up 4	patch	rivastigmine	15	83	98	84.7

*Abbreviations*: *DDD *defined daily dose

1 DDD is equal to donepezil 7.5 mg, oral rivastigmine 9 mg, rivastigmine patch 9.5 mg or galantamine 16 mg

### ChEIs, memantine, and MMSE trajectories

**Fig. 2:**
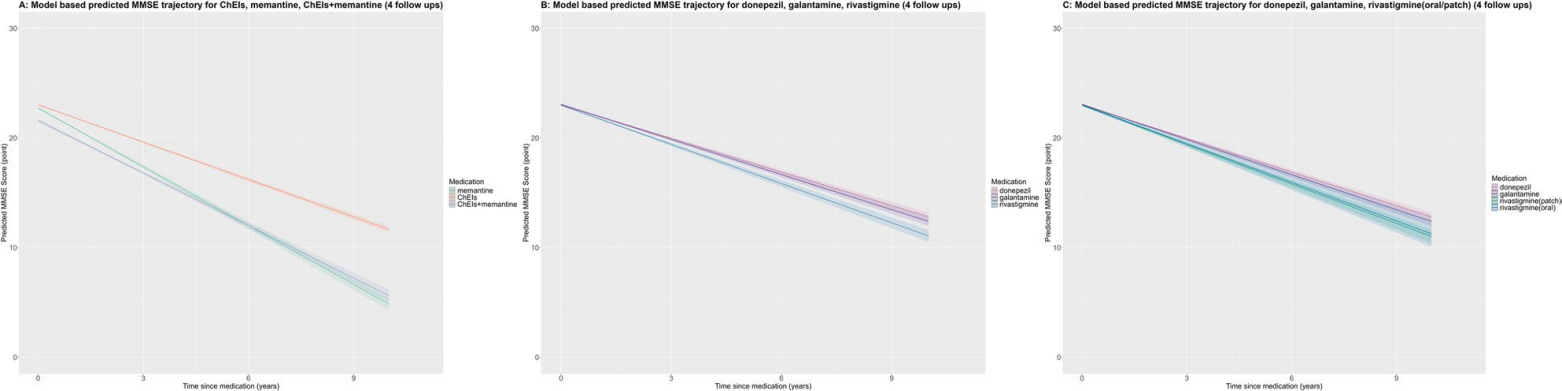
Predicted MMSE trajectories by treatment status: **A**) Predicted MMSE trajectories of ChEIs, memantine, and ChEIs added on memantine, and **B**). Predicted MMSE trajectories of donepezil, galantamine, and rivastigmine. **C**). Predicted MMSE trajectories of donepezil, galantamine, rivastigmine (oral) and rivastigmine (patch). ChEIs and ChEIs added on memantine slow down the decrease of MMSE score compared to memantine alone. Of ChEIs, donepezil and galantamine slow down the decrease of MMSE score compared to rivastigmine. No difference in the effect on MMSE decline was observed between oral and patch formulations of rivastigmine. The estimation is obtained from a cohort with inverse probability of censoring weighting (IPCW, we considered the potential effects of general attrition from those lost to follow-up due to drop-out or to the presence of a competing risk of death. The model was weighted for the following covariates: sex, age at diagnoses, calendar year of diagnosis, MMSE score at diagnosis, comorbidities (myocardial infarction, congestive heart failure, peripheral vascular disease, cerebrovascular disease, chronic obstructive pulmonary disease, chronic other pulmonary disease, rheumatic disease, hemiplegia, diabetes without chronic complication, diabetes with chronic complication, renal disease, mild liver disease, severe liver disease, peptic ulcer disease, malignancy, metastatic solid tumors, AIDS), hypertension, atrial fibrillation, presence of ongoing medications (anxiolytics, stains, antipsychotics, antidepressants, angiotensin-converting enzyme inhibitors/angiotensin receptor blockers (ACEI/ARBs), β-blockers, calcium channel blocker). Abbreviations: MMSE: Mini-Mental State Examination; ChEIs: cholinesterase inhibitors

After adjusting for all the covariates, Memantine alone users experienced a yearly cognitive decline of 1.79 MMSE points (95% CI: −1.85, − 1.73). Compared with memantine users, patients on ChEIs alone declined 0.65 points less per year (95% CI: −0.59, − 0.72), while those on combination therapy declined 0.19 points less per year (95% CI: −0.10, − 0.28). (Fig. 2A and Supplementary Table 4).

Of the ChEIs, after adjusting for all the covariates, donepezil users experienced an annual decline of 1.02 points (95% CI: −1.06, − 0.98). In comparison, galantamine users declined an additional 0.05 points per year (95% CI: −0.11, 0.01), and rivastigmine users declined an additional 0.17 points per year (95% CI: −0.24, − 0.11), relative to donepezil. (Supplementary Table 5, the predicted trajectories of MMSE score for different treatments are shown in Fig. 2B). No difference was observed between oral rivastigmine (additional decline of 0.16 points per year, 95% CI: −0.29, − 0.03) and the rivastigmine patch (additional decline of 0.18 points per year, 95% CI: −0.25, − 0.10), as measured by MMSE. (Supplementary Table 5, Fig. 2C)

While the estimates are statistically distinct, they should not be interpreted as clinically meaningful differences.

### Sensitivity analysis and subgroup analysis

When including two follow ups or seven follow ups, the results remained consistent with our main results. (Supplementary Tables 6–11, Supplementary Fig. 2–4).

Subgroup analyses revealed the consistent cognitive benefits associated with ChEIs, irrespective of age, sex, or dementia type, as indicated by the MMSE trajectories (Supplementary Fig. 5). Specifically, among patients aged over 78, users of memantine added to ChEIs showed slower cognitive decline than memantine alone user compared to patients under 78 years old. Among MxD patients, those receiving combination therapy experienced a slower rate of cognitive decline compared to those treated with memantine alone. In contrast, among AD patients, combination therapy showed little additional benefit over memantine monotherapy. The very mild and mild baseline groups showed results consistent with the primary analysis. In contrast, in the moderate and severe groups, memantine users progressed slower compared to combination therapy, but still faster than ChEIs (Supplemental Fig. 6).

## Discussion

This study provides important insights into the long-term prescription patterns and cognitive trajectories of ChEIs and memantine in a large cohort of patients with AD or MxD followed up to 11 years. The findings highlight several key observations regarding the differential cognitive trajectories of these medications and evolving treatment patterns over time.

### Prescription patterns of ChEIs and memantine

Within six months from the dementia diagnosis date, most patients (78.4%) were prescribed ChEIs, with donepezil being the most common, followed by galantamine and rivastigmine. Memantine alone was initiated in 21.6% of patients, reflecting its role as an alternative treatment option. Over time, there was a notable shift in medication use, with a steady decrease in the proportion of patients on monotherapy, particularly ChEIs alone, as many transitioned to combination therapy with ChEIs and memantine. This shift is consistent with clinical practice, where the combination of ChEIs and memantine has been increasingly adopted due to its perceived synergistic benefits in slowing cognitive decline [23, 24]. 

Interestingly, the proportion of galantamine users increased over the follow-up period, while donepezil use declined and rivastigmine remained relatively stable. This trend suggests a growing preference for galantamine, potentially due to its dual cholinergic and nicotinic receptor-modulating effects, which may offer additional cognitive benefits [25]. Notably, this observation aligns with clinical practice in Sweden, where donepezil is typically prescribed as a first-line treatment. As patients transition from initial therapy, a reduction in donepezil use is expected, with galantamine increasingly utilized to fill this therapeutic role during follow-up. Among rivastigmine users, there was a modest decrease in the use of the patch formulation and a slight increase in oral rivastigmine use, nevertheless, the proportion of patch users are much higher than oral users. This likely reflects the patch’s improved tolerability and ease of use compared to the oral form, which is often associated with gastrointestinal side effects [26]. 

The prescription patterns of ChEIs show variation in the time it takes patients to reach the defined daily dose (DDD). Donepezil and galantamine had the highest rates of patients achieving 1 DDD during follow-up, increasing from 4.5% to 81.2% and 7.9% to 91.7%, respectively. In contrast, none of the patients on oral rivastigmine reached 1 DDD at any point, suggesting poor tolerability or preference for lower doses. However, the rivastigmine patch showed better results, with 84.7% reaching 1 DDD during follow-up. These patterns suggest that delivery methods and tolerability are key factors in achieving therapeutic doses.

### Cognitive trajectories of ChEIs and memantine

Our MMSE-based trajectories reflect population-level patterns rather than individual clinical responses and estimates at the extremes should be interpreted with caution given survivor bias and limited data. Patients on ChEIs alone showed slower cognitive decline than those on memantine, or on combination therapy, though combination therapy appeared somewhat more favorable than memantine alone [27]. These exploratory findings suggest differences in population-level trajectories, but do not establish causal or clinically meaningful superiority of one regimen over another.

This could be due to the different mechanisms of action, where ChEIs increase acetylcholine levels, which is beneficial in the early stages, while memantine’s role in modulating glutamate may not provide additional benefits in these stages [8, 23]. Furthermore, studies have shown that memantine is more effective in moderate to severe stages of dementia, which might explain the lack of added cognitive protection when used alongside ChEIs in earlier stages [28, 29]. 

In fact, patients on ChEIs combined with memantine showed a faster decline compared to ChEIs alone, but a slower decline compared to memantine alone. Memantine alone was associated with the steepest cognitive decline, underscoring its more limited role in cognitive preservation compared to ChEIs. This aligns with previous studies suggesting that memantine is most effective in moderate-to-severe stages of dementia, rather than in early or mild cognitive impairment [28]. 

Memantine has also been shown to have effects on non-cognitive symptoms of dementia. It can help reduce agitation, aggression, and psychotic symptoms such as hallucinations and delusions [29]. These benefits are particularly important in managing the overall well-being and quality of life for patients with AD.

### Differential trajectories of ChEIs

There are few studies comparing the long-term associations of different ChEIs on cognition [15]. We here showed that among individual ChEIs, donepezil and galantamine were associated with slightly slower declines than rivastigmine. While this pattern is consistent with prior studies, our results should be interpreted as descriptive trends rather than evidence of differential efficacy, particularly given challenges with dosing tolerability in routine practice. These findings are consistent with prior research suggesting that donepezil may offer greater efficacy due to its more potent and selective inhibition of acetylcholinesterase [30, 31]. Galantamine’s dual mechanism, involving both cholinesterase inhibition and nicotinic receptor modulation, may also contribute to its relatively favorable cognitive outcomes [30, 31]. 

Rivastigmine, while effective, often faces challenges in reaching adequate therapeutic doses due to its side effect profile. Common adverse effects such as nausea, vomiting, and weight loss can limit dose escalation, leading to suboptimal dosing in some patients [32]. This can result in less pronounced cognitive benefits compared to donepezil and galantamine. Ensuring patients can tolerate higher doses of rivastigmine is crucial for maximizing its therapeutic potential, but this often requires careful management and gradual dose titration.

### Rivastigmine: oral vs. patch

Another interesting finding in this study cognitive trajectories did not differ substantially between oral and transdermal rivastigmine, suggesting that the route of administration does not strongly influence outcomes. Both forms showed less favorable trajectories than donepezil and galantamine, but these findings remain exploratory and descriptive of prescribing patterns and population averages. This supports the clinical use of the rivastigmine patch as an alternative to oral administration, particularly in patients who experience adverse gastrointestinal effects with oral rivastigmine [33]. However, it is important to note that the cognitive outcomes for both forms of rivastigmine were less favorable compared to those for donepezil and galantamine. This indicates that while the rivastigmine patch offers a more tolerable and convenient option without compromising its own efficacy, it may still be less effective overall in slowing cognitive decline compared to donepezil and galantamine.

Our real-world findings are broadly consistent with shorter term results from RCTs of ChEIs and memantine. Clinical trials generally report modest cognitive benefits over 6 months, with small differences among donepezil, rivastigmine, and galantamine, aligning with our observation that trajectories for these drugs are broadly similar. The strength of our study is showing the persistence of these findings over longer periods of time. Memantine trials indicate benefit primarily in moderate to severe dementia, and although our study did not include a direct comparison to untreated patients, the exploratory trajectories observed among memantine users are consistent with these trial findings [34, 35]. Overall, our results support the notion that while ChEI treatment may modestly slow cognitive decline long-term, differences between the ChEI drugs are small and should be interpreted in the context of real-world clinical practice and patient heterogeneity.

### Implications and future directions

This study provides valuable insights into the real-world treatment patterns and cognitive trajectories of patients with AD or MxD over an extended period. While the data do not allow for conclusions about comparative effectiveness due to the observational design and lack of untreated controls, the findings reflect current clinical practice and can inform future research and therapeutic strategies.

The observed patterns of ChEI use—particularly the increased use of galantamine and stable use of rivastigmine—underscore the importance of individualized treatment decisions based on patient tolerability, preferences, and clinical characteristics. Donepezil and galantamine were the most initiated ChEIs, while rivastigmine patches appeared to offer a tolerable alternative to oral formulations.

Although memantine-treated patients showed different cognitive trajectories compared to those on ChEIs, these differences likely reflect underlying disease severity and treatment indications rather than drug efficacy. Memantine may still play a role in managing non-cognitive symptoms, especially in later stages of dementia, but further research is needed to clarify its long-term impact.

Importantly, this study highlights the need for future investigations using causal inference methods, such as target trial emulation, to better understand treatment effects while accounting for confounding by indication. Additionally, the integration of ChEIs with emerging disease-modifying therapies, such as anti-amyloid antibodies (e.g., lecanemab, donanemab), represents a promising area for future exploration[36–38]. These combinations may offer synergistic benefits by targeting both symptomatic and pathological aspects of AD.

Finally, the longitudinal data from this large, well-characterized cohort can serve as a historical control for evaluating the effectiveness of new therapies. Understanding the natural history of cognitive decline under standard care is essential for contextualizing outcomes in future interventional studies.

### Strengths and limitations

This study, based on SveDem, one of the largest dementia cohorts of its kind[15, 39], adds valuable insights into the long-term effectiveness of ChEIs and memantine in a large cohort in real-world settings. It further explores prescription patterns and the attainment of defined daily doses (DDD). These findings can help guide clinical decisions to improve cognitive outcomes and quality of life for dementia patients.

Several limitations should be noted. First, the absence of randomization introduces the possibility of treatment allocation bias and confounding by indication, as prescribing decisions are influenced by patient characteristics such as cognitive status and comorbidities[40]. Second, while findings are generalizable within the SveDem population, extrapolation to other, more homogeneous populations should be done with caution. Third, not all relevant confounders could be addressed, including socioeconomic and lifestyle factors that may affect cognition. Fourth, although the study includes follow-up of up to 11 years, not all patients were followed for this duration, limiting the ability to fully capture long-term trajectories. Moreover, given the large sample size and multiple comparisons, some statistically significant findings may represent false positives or reflect very small, clinically negligible differences.

Methodologically, our analyses do not fully account for dynamic treatment changes, as patients may initiate, discontinue, or switch therapies after baseline. While our descriptive approach was intended to provide exploratory insights, more advanced causal inference methods-such as the parametric g-formula-would be better suited to rigorously address time-varying exposures and confounding, and we highlight this as an important direction for future research[41]. Finally, our requirement that patients survive at least six months after diagnosis to ascertain medication use and enable longitudinal MMSE follow-up may have introduced survivor bias, excluding individuals with poor short-term prognosis and limiting generalizability to healthier subgroups.

Taken together, these limitations emphasize that our results should be interpreted as hypothesis-generating and descriptive of real-world practice, rather than as definitive evidence of causal treatment effects.

## Conclusions

In this large cohort of patients with AD or MxD, ChEIs users alone, particularly donepezil and galantamine, showed slower cognitive decline compared to users of memantine or combination therapy users. While differences were modest, the results contribute to a better understanding of treatment trajectories in routine clinical practice. The study highlights evolving prescription patterns and the need for individualized treatment based on cognitive effects and patient tolerability, while further research is needed to optimize dementia care strategies.

## Supplementary Information

Supplementary Material 1.

## Data Availability

The datasets used and/or analysed during the current study are available from the Swedish registry for cognitive/dementia disorders.
