# Supplementary material for: Dynamic associations of cholinesterase inhibitors and memantine with cognitive trajectories in individuals with Alzheimer’s or mixed dementia: a real-world analysis using the quality registry SveDem
Source: Alzheimers Res Ther. 2025 Nov 28;17:256. doi: 10.1186/s13195-025-01918-0 (PMC12670791; doi:10.1186/s13195-025-01918-0)
Supplement: Supplementary file 1 — Supplementary Material 1. [file 13195_2025_1918_MOESM1_ESM.docx]

**Supplementary Figure 1.** Flowchart

Abbreviations: ChEIs, cholinesterase inhibitors; MMSE, Mini-Mental State Examination.

**Supplementary Figure 2.** Sequential Sankey plot by treatment status for patients with at most 4 follow ups: A) Memantine, ChEIs, and ChEIs add on memantine, B) Donepezil, galantamine, rivastigmine, C) Donepezil, galantamine, rivastigmine (oral) and rivastigmine (patch).

Abbreviations: ChEIs: cholinesterase inhibitors.





**Supplementary Figure 3.** Predicted MMSE trajectories by treatment status for patients with up to 2 follow ups (maximum follow up time 10 years): A) Predicted MMSE trajectories of ChEIs, memantine, and ChEIs added on memantine, and B). Predicted MMSE trajectories of donepezil, galantamine, and rivastigmine. C). Predicted MMSE trajectories of donepezil, galantamine, rivastigmine (oral) and rivastigmine (patch). ChEIs and ChEIs added on memantine slow down the decrease of MMSE score compared to memantine alone. Of ChEIs, donepezil and galantamine slow down the decrease of MMSE score compared to rivastigmine. No significant difference in the effect on MMSE decline was observed between oral and patch formulations of rivastigmine.

Abbreviations: ChEIs: cholinesterase inhibitors; MMSE: Mini-Mental State Examination.





**Supplementary Figure 4.** Sankey plot by treatment status for patients with up to 7 follow ups (maximum follow up time 13 years): A) Memantine, ChEIs, and ChEIs add on memantine, B) Donepezil, galantamine, rivastigmine, C) Donepezil, galantamine, rivastigmine (oral) and rivastigmine (patch).

Abbreviations: ChEIs: cholinesterase inhibitors.

Notes: 0 (baseline, within six months after dementia diagnosis date), 1 (within six months after first follow up), 2 (within six months after second follow up), 3 (within six months after third follow up), 4 (within six months after fourth follow up), 5 (within six months after fifth follow up), 6 (within six months after sixth follow up), 7 (within six months after seventh follow up).

**

**

**Supplementary Figure 5.** Predicted MMSE trajectories by treatment status for patients with up to 7 follow ups (maximum follow up time 13 years): A) Predicted MMSE trajectories of ChEIs, memantine, and ChEIs added on memantine, and B). Predicted MMSE trajectories of donepezil, galantamine, and rivastigmine. C). Predicted MMSE trajectories of donepezil, galantamine, rivastigmine (oral) and rivastigmine (patch). ChEIs and ChEIs added on memantine slow down the decrease of MMSE score compared to memantine alone. Of ChEIs, donepezil and galantamine slow down the decrease of MMSE score compared to rivastigmine. No significant difference in the effect on MMSE decline was observed between oral and patch formulations of rivastigmine.

Abbreviations: ChEIs: cholinesterase inhibitors; MMSE: Mini-Mental State Examination.





**Supplementary Figure 6.** Predicted MMSE trajectories by treatment status for subgroup patients with up to 4 follow ups (maximum follow ups time 11 years): A) Predicted MMSE trajectories of ChEIs, memantine, and ChEIs added on memantine for male patients, and B) Predicted MMSE trajectories of ChEIs, memantine, and ChEIs added on memantine for female patients, and C) Predicted MMSE trajectories of ChEIs, memantine, and ChEIs added on memantine for patients age <= 78, and D) Predicted MMSE trajectories of ChEIs, memantine, and ChEIs added on memantine for patients age > 78, and E) Predicted MMSE trajectories of ChEIs, memantine, and ChEIs added on memantine for AD patients, and F) Predicted MMSE trajectories of ChEIs, memantine, and ChEIs added on memantine for MxD patients, and G) Predicted MMSE trajectories of ChEIs, memantine, and ChEIs added on memantine for very mild patients (baseline MMSE≥25), and H) Predicted MMSE trajectories of ChEIs, memantine, and ChEIs added on memantine for mild patients (baseline MMSE 20-24), and I) Predicted MMSE trajectories of ChEIs, memantine, and ChEIs added on memantine for moderate patients (baseline MMSE 10-19), and J) Predicted MMSE trajectories of ChEIs, memantine, and ChEIs added on memantine for severe patients(baseline MMSE ≤9). Overall, ChEIs and ChEIs added on memantine slow down the decrease of MMSE score compared to memantine alone.

Abbreviations: AD: Alzheimer's disease; MxD: mixed dementia; ChEIs: cholinesterase inhibitors; MMSE: Mini-Mental State Examination.





**Supplementary Table 1. ICD 10 codes and ATC codes of comorbidities and medications.**

| **Comorbidities** | **ICD 10 codes** |  |
| --- | --- | --- |
| Myocardial infarction | I21, I22, I252 |  |
| Congestive heart failure | I110, I130, I132, I255, I420, I426-I429, I43, I50 |  |
| Peripheral vascular disease | I70, I71, I731, I738, I739, I771, I790, I792, K55 |  |
| Cerebrovascular disease | G45, I60-I64, I67, I69 |  |
| Chronic obstructive pulmonary disease | J43, J44 |  |
| Chronic other pulmonary disease | J41, J42, J45-J47, J60-J70 |  |
| Rheumatic disease | M05, M06, M123, M070-M073, M08, M13, M30, M313-M316, M32-M34, M350, M351, M353, M45, M46 |  |
| Hemiplegia | G114, G80-G82, G830-G833, G838 |  |
| Diabetes without chronic complication | E100, E101, E110, E111, E120, E121, E130, E131, E140, E141 |  |
| Diabetes with chronic complication | E102-E105, E107, E112-E117, E122-E127, E132-E137, E142-E147 |  |
| Renal disease | I120, I131, N032-N037, N052-N057, N11, N18, N19, N250, Q611-Q614, Z49, Z940, Z992 |  |
| Mild liver disease | B15-B19, K703, K709, K73, K746, K754 |  |
| Severe liver disease | I850, I859, I982, I983 |  |
| Peptic ulcer disease | K25-K28 |  |
| Malignancy | C00-C41, C43, C45-C58, C60-C76, C81-C86, C88-C97 |  |
| Metastatic solid tumors | C77-C80 |  |
| AIDS | B20-B24, F024, O987, R75, Z219, Z717 |  |
| Hypertension | I10-I15 |  |
| Atrial fibrillation | I48 |  |
| **Medication** |  | **ATC codes** |
| Anxiolytics |  | N05B |
| Statins |  | C10 |
| Antipsychotics |  | N05A |
| Antidepressants |  | N06A |
| ACEI/ARBs |  | C09 |
| Beta blockers |  | C07 |
| Calcium channel blocker |  | C08 |
| Anti-dementia drugs |  |  |
| Memantine |  | N06DX01 |
| Donepezil |  | N06DA02 |
| Rivastigmine |  | N06DA03 |
| Galantamine |  | N06DA04 |

Abbreviations: ACEI, angiotensin-converting enzyme inhibitors; ARB, angiotensin receptor blockers.

**Supplementary table 2. Medication status within six months after dementia diagnosis date and 4 follow-ups for ChEIs, memantine, and memantine added to ChEIs.**

|  | Number of follow-ups (baseline= within six months after dementia diagnosis date, 1= within six months after the first follow up, 2= within six months after the second follow up, 3= within six months after the third follow up, 4= within six months after the fourth follow up) | | | | |
| --- | --- | --- | --- | --- | --- |
| Characteristics | baseline | 1 | 2 | 3 | 4 |
| Number of patients | 32282 | 13745 | 5641 | 2663 | 1270 |
| MMSE (mean (SD)) | 21.4 (4.7) | 21.1 (4.8) | 20.1 (5.2) | 19.4 (5.5) | 19.4 (5.6) |
| Follow up time / year  (mean (SD)) | 0.0 (0.0) | 1.0 (0.6) | 2.0 (0.8) | 3.1 (1.0) | 4.0 (1.1) |
| Medication (%) |  |  |  |  |  |
| ChEIs | 25318 (78.4) | 9467 (68.9) | 3356 (59.5) | 1402 (52.6) | 665 (52.4) |
| Memantine | 6964 (21.6) | 2544 (18.5) | 862 (15.3) | 403 (15.1) | 178 (14.0) |
| ChEIs+memantine | 0 (0.0) | 1734 (12.6) | 1423 (25.2) | 858 (32.2) | 427 (33.6) |
| Drop out |  |  |  |  |  |
| Death | 0 | 11093 | 5188 | 1731 | 737 |
| Loss of follow up | 0 | 7444 | 2916 | 1247 | 656 |

Abbreviations: MMSE: Mini-Mental State Examination; SD: standard deviation, ChEIs: cholinesterase inhibitors.

**Supplementary table 3. Medication status within six months after dementia diagnosis date and 4 follow-ups for ChEIs.**

|  | Number of follow-ups (baseline= within six months after dementia diagnosis date, 1= within six months after the first follow up, 2= within six months after the second follow up, 3= within six months after the third follow up, 4= within six months after the fourth follow up) | | | | |
| --- | --- | --- | --- | --- | --- |
| Characteristics | baseline | 1 | 2 | 3 | 4 |
| Number of patients | 25 318 | 9447 | 3350 | 1401 | 664 |
| MMSE (mean (SD)) | 21.9 (4.4) | 22.0 (4.4) | 21.3 (4.8) | 20.7 (5.2) | 20.9 (5.2) |
| Follow up time / year  (mean (SD)) | 0.0 (0.0) | 0.9 (0.6) | 2.0 (0.8) | 3.1 (1.1) | 4.1 (1.2) |
| Medication (%) |  |  |  |  |  |
| Donepezil  average follow up time/ year | 15 266 (60.3)  0 | 5466 (57.9)  0.9 | 1884 (56.2)  2 | 761 (54.3)  3.1 | 362 (54.5)  4 |
| Galantamine  average follow up time/ year | 5217 (20.6)  0 | 2236 (23.7)  0.9 | 840 (25.1)  2 | 357 (25.5)  3.2 | 168 (25.3)  4.4 |
| Rivastigmine  average follow up time/ year | 4835 (19.1)  0 | 1745 (18.5)  0.9 | 626 (18.7)  2 | 283 (20.2)  3 | 134 (20.2)  4.1 |
| Rivastigmine (patch)  average follow up time/ year | 4169 (16.5)  0 | 1468 (15.5)  0.9 | 493 (14.7)  2 | 214 (15.3)  3 | 98 (14.8)  4 |
| Rivastigmine (oral)  average follow up time/ year | 666 (2.6)  0 | 277 (2.9)  1.1 | 133 (4.0)  2.1 | 69 (4.9)  3 | 36 (5.4)  4.2 |

Abbreviations: MMSE: Mini-Mental State Examination; SD: standard deviation, ChEIs: cholinesterase inhibitors.

**Supplementary table 4. Estimated MMSE trajectories by treatment status (****memantine, ChEIs) with all covariates adjusted (4 follow ups).**

| Treatment status | MMSE score estimation, β coefficient (95% CI) | | |
| --- | --- | --- | --- |
|  | Memantine | ChEIs | ChEIs+memantine |
| At diagnosis | 22.7 (22.7,22.8) | 23 (23,23.1) | 21.6 (21.4,21.7) |
| Year 1 | 20.9 (20.9,21) | 21.9 (21.8,22) | 20 (19.9,20.1) |
| Year 3 | 17.4 (17.2,17.5) | 19.6 (19.5,19.7) | 16.8 (16.7,16.9) |
| Year 5 | 13.8 (13.5,14.1) | 17.4 (17.2,17.5) | 13.6 (13.4,13.8) |
| MMSE slope  points/year | -1.79 (-1.85, -1.73) | -1.14 (-1.20, -1.07) | -1.60 (-1.69. -1.51) |

Note: The estimation is obtained from a cohort with inverse probability of censoring weighting (IPCW, we considered the potential effects of general attrition from those lost to follow-up due to drop-out or to the presence of a competing risk of death. The model was weighted for the following covariates: sex, age at diagnoses, calendar year of diagnosis, MMSE score at diagnosis, comorbidities (myocardial infarction, congestive heart failure, peripheral vascular disease, cerebrovascular disease, chronic obstructive pulmonary disease, chronic other pulmonary disease, rheumatic disease, hemiplegia, diabetes without chronic complication, diabetes with chronic complication, renal disease, mild liver disease, severe liver disease, peptic ulcer disease, malignancy, metastatic solid tumors, AIDS), hypertension, atrial fibrillation, presence of ongoing medications (anxiolytics, stains, antipsychotics, antidepressants, angiotensin-converting enzyme inhibitors /angiotensin receptor blockers (ACEI/ARBs), β-blockers, calcium channel blocker).

Abbreviations: MMSE: Mini-Mental State Examination; CI: confidence interval; ChEIs: cholinesterase inhibitors.

**Supplementary table 5. Estimated MMSE trajectories by treatment status (donepezil, galantamine, rivastigmine) with all covariates adjusted (4 follow ups).**

| Treatment status | MMSE score estimation, β coefficient (95% CI) | | | | |
| --- | --- | --- | --- | --- | --- |
|  | Donepezil | Galantamine | Rivastigmine | Rivastigmine(oral) | Rivastigmine(patch) |
| At diagnosis | 23 (23,23.1) | 23.1 (23,23.1) | 23 (22.9,23.1) | 23 (22.9,23.2) | 23 (22.9,23.1) |
| Year 1 | 22 (21.9,22.1) | 22 (21.9,22.1) | 21.8 (21.7,21.9) | 21.8 (21.7,22) | 21.8 (21.7,21.9) |
| Year 3 | 20 (19.8,20.1) | 19.9 (19.7,20) | 19.4 (19.3,19.6) | 19.5 (19.2,19.8) | 19.4 (19.2,19.6) |
| Year 5 | 17.9 (17.7,18.1) | 17.7 (17.5,17.9) | 17 (16.8,17.3) | 17.1 (16.6,17.7) | 17 (16.7,17.3) |
| MMSE slope  points/year | -1.02 (-1.06, -0.98) | -1.07 (-1.13, -1.01) | -1.19 (-1.26, -1.13) | -1.18 (-1.31, -1.05) | -1.20 (-1.27, -1.12) |

Note: The estimation is obtained from a cohort with inverse probability of censoring weighting (IPCW, we considered the potential effects of general attrition from those lost to follow-up due to drop-out or to the presence of a competing risk of death. The model was weighted for the following covariates: sex, age at diagnoses, calendar year of diagnosis, MMSE score at diagnosis, comorbidities (myocardial infarction, congestive heart failure, peripheral vascular disease, cerebrovascular disease, chronic obstructive pulmonary disease, chronic other pulmonary disease, rheumatic disease, hemiplegia, diabetes without chronic complication, diabetes with chronic complication, renal disease, mild liver disease, severe liver disease, peptic ulcer disease, malignancy, metastatic solid tumors, AIDS), hypertension, atrial fibrillation, presence of ongoing medications (anxiolytics, stains, antipsychotics, antidepressants, angiotensin-converting enzyme inhibitors /angiotensin receptor blockers (ACEI/ARBs), β-blockers, calcium channel blocker).

Abbreviations: MMSE: Mini-Mental State Examination; CI: confidence interval; ChEIs: cholinesterase inhibitors.

**Supplementary table 6. Estimated MMSE trajectories by treatment status (memantine, ChEIs) with all covariates adjusted (2 follow ups).**

| Treatment status | MMSE score estimation, β coefficient (95% CI) | | |
| --- | --- | --- | --- |
|  | Memantine | ChEIs | ChEIs+memantine |
| At diagnosis | 21.8 (21.8,21.9) | 22.1 (22,22.1) | 20.9 (20.7,21) |
| Year 1 | 19.9 (19.8,20) | 20.9 (20.9,21) | 19.1 (19,19.2) |
| Year 3 | 16 (15.8,16.2) | 18.7 (18.6,18.8) | 15.5 (15.3,15.7) |
| Year 5 | 12.1(11.8,12.5) | 16.4 (16.2,16.5) | 11.9 (11.5,12.3) |
| MMSE slope  points/year | -1.94 (-2.01, -1.87) | -1.14 (-1.22, -1.06) | -1.79 (-1.91, -1.66) |

Note: The estimation is obtained from a cohort with inverse probability of censoring weighting (IPCW, we considered the potential effects of general attrition from those lost to follow-up due to drop-out or to the presence of a competing risk of death. The model was weighted for the following covariates: sex, age at diagnoses, calendar year of diagnosis, MMSE score at diagnosis, comorbidities (myocardial infarction, congestive heart failure, peripheral vascular disease, cerebrovascular disease, chronic obstructive pulmonary disease, chronic other pulmonary disease, rheumatic disease, hemiplegia, diabetes without chronic complication, diabetes with chronic complication, renal disease, mild liver disease, severe liver disease, peptic ulcer disease, malignancy, metastatic solid tumors, AIDS), hypertension, atrial fibrillation, presence of ongoing medications (anxiolytics, stains, antipsychotics, antidepressants, angiotensin-converting enzyme inhibitors /angiotensin receptor blockers (ACEI/ARBs), β-blockers, calcium channel blocker).

Abbreviations: MMSE: Mini-Mental State Examination; CI: confidence interval; ChEIs: cholinesterase inhibitors.

**Supplementary table 7. Estimated MMSE trajectories by treatment status (donepezil, galantamine, rivastigmine) with all covariates adjusted (2 follow ups).**

| Treatment status | MMSE score estimation, β coefficient (95% CI) | | | | |
| --- | --- | --- | --- | --- | --- |
|  | Donepezil | Galantamine | Rivastigmine | Rivastigmine(oral) | Rivastigmine(patch) |
| At diagnosis | 23 (23,23.1) | 23.1 (23,23.1) | 23 (22.9,23.1) | 23 (22.9,23.2) | 23.0 (22.9,23.1) |
| Year 1 | 22 (22,22.1) | 22 (22,22.1) | 21.7 (21.6,21.8) | 21.8 (21.7,22) | 21.7 (21.6,21.8) |
| Year 3 | 20 (19.9,20.2) | 20 (19.8,20.1) | 19.1 (19,19.3) | 19.4 (19,19.8) | 19.1 (18.9,19.3) |
| Year 5 | 18 (17.8,18.2) | 17.9 (17.7,18.2) | 16.6 (16.3,16.9) | 16.9 (16.2,17.6) | 16.5(16.1,16.8) |
| MMSE slope  points/year | -1.00 (-1.05, -0.95) | -1.03 (-1.10, -1.04) | -1.29 (-1.37, -1.20) | -1.22 (-1.38, -1.07) | -1.30 (-1.39, -1.22) |

Note: The estimation is obtained from a cohort with inverse probability of censoring weighting (IPCW, we considered the potential effects of general attrition from those lost to follow-up due to drop-out or to the presence of a competing risk of death. The model was weighted for the following covariates: sex, age at diagnoses, calendar year of diagnosis, MMSE score at diagnosis, comorbidities (myocardial infarction, congestive heart failure, peripheral vascular disease, cerebrovascular disease, chronic obstructive pulmonary disease, chronic other pulmonary disease, rheumatic disease, hemiplegia, diabetes without chronic complication, diabetes with chronic complication, renal disease, mild liver disease, severe liver disease, peptic ulcer disease, malignancy, metastatic solid tumors, AIDS), hypertension, atrial fibrillation, presence of ongoing medications (anxiolytics, stains, antipsychotics, antidepressants, angiotensin-converting enzyme inhibitors /angiotensin receptor blockers (ACEI/ARBs), β-blockers, calcium channel blocker).

Abbreviations: MMSE: Mini-Mental State Examination; CI: confidence interval; ChEIs: cholinesterase inhibitors.

**Supplementary table 8. Medication status within six months after dementia diagnosis date and 7 follow-ups for ChEIs, memantine, and ChEIs added on memantine.**

|  | Number of follow-ups (baseline= within six months after dementia diagnosis date, 1= within six months after the first follow up, 2= within six months after the second follow up, 3= within six months after the third follow up, 4= within six months after the fourth follow up, 5= within six months after the fifth follow up, 6= within six months after the sixth follow up, 7= within six months after the seventh follow up) | | | | | | | |
| --- | --- | --- | --- | --- | --- | --- | --- | --- |
| Characteristics | baseline | 1 | 2 | 3 | 4 | 5 | 6 | 7 |
| Number of patients | 32282 | 13745 | 5641 | 2663 | 1270 | 598 | 261 | 136 |
| MMSE (mean (SD)) | 21.4 (4.7) | 21.1 (4.8) | 20.1 (5.2) | 19.4 (5.5) | 19.4 (5.6) | 19.2 (5.9) | 19.6 (5.6) | 19.3 (6.1) |
| Follow up time / year  (mean (SD)) | 0.0 (0.0) | 1.0 (0.6) | 2.0 (0.8) | 3.1 (1.0) | 4.0 (1.1) | 5.2 (1.4) | 6.2 (1.4) | 7.0 (1.2) |
| Medication (%) |  |  |  |  |  |  |  |  |
| ChEIs | 25318 (78.4) | 9467 (68.9) | 3356 (59.5) | 1402 (52.6) | 665 (52.4) | 295 (49.3) | 137 (52.5) | 72 (52.9) |
| Memantine | 6964 (21.6) | 2544 (18.5) | 862 (15.3) | 403 (15.1) | 178 (14.0) | 84 (14.0) | 30 (11.5) | 14 (10.3) |
| ChEIs+memantine | 0 (0.0) | 1734 (12.6) | 1423 (25.2) | 858 (32.2) | 427 (33.6) | 219 (36.6) | 94 (36.0) | 50 (36.8) |

Abbreviations: MMSE: Mini-Mental State Examination; SD: standard deviation, ChEIs: cholinesterase inhibitors.

**Supplementary table 9. Medication status within six months after dementia diagnosis date and 7 follow-ups for ChEIs.**

|  | Number of follow-ups (baseline= within six months after dementia diagnosis date, 1= within six months after the first follow up, 2= within six months after the second follow up, 3= within six months after the third follow up, 4= within six months after the fourth follow up, 5= within six months after the fifth follow up, 6= within six months after the sixth follow up, 7= within six months after the seventh follow up) | | | | | | | |
| --- | --- | --- | --- | --- | --- | --- | --- | --- |
| Characteristics | baseline | 1 | 2 | 3 | 4 | 5 | 6 | 7 |
| Number of patients | 25 318 | 9447 | 3350 | 1401 | 664 | 295 | 137 | 72 |
| MMSE (mean (SD)) | 21.9 (4.4) | 22.0 (4.4) | 21.3 (4.8) | 20.7 (5.2) | 20.9 (5.2) | 21.1 (5.5) | 21.7 (5.0) | 20.9 (5.6) |
| Follow up time / year  (mean (SD)) | 0.0 (0.0) | 0.9 (0.6) | 2.0 (0.8) | 3.1 (1.1) | 4.1 (1.2) | 5.2 (1.4) | 6.2 (1.5) | 7.1 (1.1) |
| Medication (%) |  |  |  |  |  |  |  |  |
| Donepezil  average follow up time/ year | 15 266 (60.3)  0 | 5466 (57.9)  0.9 | 1884 (56.2)  2 | 761 (54.3)  3.1 | 362 (54.5)  4 | 144 (48.8)  5.1 | 53 (38.7)  6 | 28 (38.9)  7.1 |
| Galantamine  average follow up time/ year | 5217 (20.6)  0 | 2236 (23.7)  0.9 | 840 (25.1)  2 | 357 (25.5)  3.2 | 168 (25.3)  4.4 | 85 (28.8)  5.5 | 52 (38.0)  6.6 | 30 (41.7)  7.4 |
| Rivastigmine  average follow up time/ year | 4835 (19.1)  0 | 1745 (18.5)  0.9 | 626 (18.7)  2 | 283 (20.2)  3 | 134 (20.2)  4.1 | 66 (22.4)  5.2 | 32 (23.4)  6.1 | 14 (19.4)  6.4 |
| Rivastigmine (patch)  average follow up time/ year | 4169 (16.5)  0 | 1468 (15.5)  0.9 | 493 (14.7)  2 | 214 (15.3)  3 | 98 (14.8)  4 | 47 (15.9)  5.1 | 26 (19.0)  5.8 | 11 (15.3)  6.6 |
| Rivastigmine (oral)  average follow up time/ year | 666 (2.6)  0 | 277 (2.9)  1.1 | 133 (4.0)  2.1 | 69 (4.9)  3 | 36 (5.4)  4.2 | 19 (6.4)  5.5 | 6 (4.4)  7.3 | 3 (4.2)  5.8 |

Abbreviations: SD: standard deviation; ChEIs: cholinesterase inhibitors.

**Supplementary table 10.** **Estimated MMSE trajectories by treatment status (memantine, ChEIs) with all covariates adjusted (7 follow ups).**

| Treatment status | MMSE score estimation, β coefficient (95% CI) | | |
| --- | --- | --- | --- |
|  | Memantine | ChEIs | ChEIs+memantine |
| At diagnosis | 22.7 (22.6,22.8) | 23.0 (23.0,23.1) | 21.4 (21.3,21.5) |
| Year 1 | 21 (20.9,21.1) | 21.9 (21.9,22) | 19.9 (19.8,20) |
| Year 3 | 17.7 (17.5,17.8) | 19.8 (19.7,19.8) | 17 (16.9,17.1) |
| Year 5 | 14.3 (14.1,14.6) | 17.6 (17.4,17.7) | 14.1 (13.9,14.3) |
| MMSE slope  points/year | -1.68 (-1.73, -1.62) | -1.09 (-1.15, -1.03) | -1.47 (-1.55, -1.39) |

Note: The estimation is obtained from a cohort with inverse probability of censoring weighting (IPCW, we considered the potential effects of general attrition from those lost to follow-up due to drop-out or to the presence of a competing risk of death. The model was weighted for the following covariates: sex, age at diagnoses, calendar year of diagnosis, MMSE score at diagnosis, comorbidities (myocardial infarction, congestive heart failure, peripheral vascular disease, cerebrovascular disease, chronic obstructive pulmonary disease, chronic other pulmonary disease, rheumatic disease, hemiplegia, diabetes without chronic complication, diabetes with chronic complication, renal disease, mild liver disease, severe liver disease, peptic ulcer disease, malignancy, metastatic solid tumors, AIDS), hypertension, atrial fibrillation, presence of ongoing medications (anxiolytics, stains, antipsychotics, antidepressants, angiotensin-converting enzyme inhibitors /angiotensin receptor blockers (ACEI/ARBs), β-blockers, calcium channel blocker).

Abbreviations: MMSE: Mini-Mental State Examination; CI: confidence interval; ChEIs: cholinesterase inhibitors.

**Supplementary table 11. Estimated MMSE trajectories by treatment status (donepezil, galantamine, rivastigmine) with all covariates adjusted (7 follow ups).**

| Treatment status | MMSE score estimation, β coefficient (95% CI) | | | | |
| --- | --- | --- | --- | --- | --- |
|  | Donepezil | Galantamine | Rivastigmine | Rivastigmine(oral) | Rivastigmine(patch) |
| At diagnosis | 23.0 (23,23.1) | 23.1 (23,23.1) | 23.0 (22.9,23) | 23.0 (22.9,23.1) | 23.0 (22.9,23) |
| Year 1 | 22.0 (22,22.1) | 22.0 (22,22.1) | 21.9 (21.8,21.9) | 21.9 (21.7,22) | 21.9 (21.8,21.9) |
| Year 3 | 20.1 (20,20.2) | 19.9 (19.8,20.1) | 19.7 (19.5,19.8) | 19.6 (19.3,19.9) | 19.7 (19.5,19.8) |
| Year 5 | 18.1 (17.9,18.3) | 17.9 (17.7,18.1) | 17.4 (17.2,17.7) | 17.4 (16.8,17.9) | 17.5 (17.2,17.7) |
| MMSE slope  points/year | -0.98 (-1.02, -0.95) | -1.04 (-1.09, -0.98) | -1.10 (-1.17, -1.04) | -1.12 (-1.24, -1.00) | -1.10 (-1.17, -1.03) |

Note: The estimation is obtained from a cohort with inverse probability of censoring weighting (IPCW, we considered the potential effects of general attrition from those lost to follow-up due to drop-out or to the presence of a competing risk of death. The model was weighted for the following covariates: sex, age at diagnoses, calendar year of diagnosis, MMSE score at diagnosis, comorbidities (myocardial infarction, congestive heart failure, peripheral vascular disease, cerebrovascular disease, chronic obstructive pulmonary disease, chronic other pulmonary disease, rheumatic disease, hemiplegia, diabetes without chronic complication, diabetes with chronic complication, renal disease, mild liver disease, severe liver disease, peptic ulcer disease, malignancy, metastatic solid tumors, AIDS), hypertension, atrial fibrillation, presence of ongoing medications (anxiolytics, stains, antipsychotics, antidepressants, angiotensin-converting enzyme inhibitors /angiotensin receptor blockers (ACEI/ARBs), β-blockers, calcium channel blocker).

Abbreviations: MMSE: Mini-Mental State Examination; CI: confidence interval.
